# Digital twin–supported behavioral intention in mothers of young children to prevent childhood obesity: a large language model–based intervention study

**DOI:** 10.3389/frai.2026.1927993

**Published:** 2026-08-06

**Authors:** Kenji Nakamura, Genki Shinoda, Aoi Noda, Mami Ishikuro, Taku Obara, Taeka Matsubara, Hideki Ishii, Masahiro Onishi, Yoshiaki Ohyama

**Affiliations:** 1 Center for Mathematics and Data Science, Gunma University, Maebashi, Gunma, Japan; 2 Innovative Medical Research Center, Gunma University Hospital, Maebashi, Gunma, Japan; 3 Tohoku Medical Megabank Organization, Tohoku University, Sendai, Miyagi, Japan; 4 Interfaculty Initiative in Information Studies, The University of Tokyo, Tokyo, Japan; 5 Department of Cardiovascular Medicine, Graduate School of Medicine, Gunma University, Maebashi, Gunma, Japan; 6 System Integration Center, Gunma University Hospital, Maebashi, Gunma, Japan

**Keywords:** behavioral intention, childhood obesity, digital twin, large language model, maternal and child health, nutrition counseling, precision prevention

## Abstract

**Introduction:**

Childhood obesity is a major determinant of lifelong non-communicable disease risk, yet early intervention may modify this trajectory. Digital twins and large language models may provide a scalable means of translating individualized risk prediction into understandable and motivating lifestyle guidance.

**Methods:**

We developed a precision-preventive intervention integrating a childhood-overweight digital twin with an empathic, supervised-fine-tuned open-source nutrition-guidance large language model. The model was based on Llama 3-70B and fine-tuned using the Standard Tables of Food Composition in Japan 2020 and its 2023 amendment. Response appropriateness was evaluated using 30 nutrition-related questions independently assessed by three registered dietitians. The digital twin adopted a published Tohoku Medical Megabank Birth and Three-Generation cohort model predicting overweight risk at 36–47 months, 6 years, 11 years, and 14 years. A total of 121 women with a child under 14 years of age participated in the digital-twin visualization and a single chat-based test.

**Results:**

The off-the-shelf Llama 3-70B produced a mean of 18 appropriate responses out of 30, whereas the fine-tuned model produced 27 out of 30. The digital-twin visualization and chat consultation were rated highly for clarity, perceived value, behavioral intention, and willingness to continue, with core-item means above 4.9 on a five-point scale. Thematic analysis showed that 112 of 121 participants perceived the recommended lifestyle changes as feasible and achievable gradually.

**Discussion:**

The integrated intervention demonstrated preliminary operational feasibility and acceptability. Individualized future-risk visualization combined with supportive conversational guidance was associated with high self-reported behavioral intention. Controlled and longitudinal studies are required to determine whether these preliminary motivational responses translate into sustained behavior change and improved health outcomes.

## 1 Introduction

### 1.1 From population medicine to precision, preventive care

Modern healthcare is undergoing a paradigm shift from population-level, one-size-fits-all medicine toward precision medicine - care tailored to the individual’s specific biological, behavioral, and environmental profile. The concept originated principally from genomics: in 2015, the United States launched a national Precision Medicine Initiative explicitly grounded in using an individual’s genomic, environmental, and lifestyle information to guide diagnosis, treatment, and prevention rather than applying uniform protocols across a population (Collins and Varmus, 2015). This genomic paradigm reframed disease as an imbalance between an individual’s own biology and their environment, and reframed prevention as a matter of estimating that individual’s specific trajectory rather than an averaged population risk.

Translating this vision into governed practice raises a consideration directly relevant to the present work: precision, data-driven interventions built on an individual’s biological and lifestyle information carry genuine social risk when governance lags behind technical capability, and their legitimacy rests not only on predictive accuracy but on how responsibly the underlying personal data is stored, controlled, and protected. Different jurisdictions have approached this at different paces - for example, the European Union treats genetic and health data as a specially protected category under the General Data Protection Regulation, while Japan enacted a dedicated Genomic Medicine Promotion Act only in 2023 (Government of Japan, 2023). Rather than surveying these frameworks in depth, we take from them a single design implication that motivates the present study: precision-preventive systems handling sensitive family health data benefit from locally controllable, vendor-independent architectures, an argument developed in Section 2.3.

### 1.2 Childhood obesity: a reversible, developmentally rooted risk

Childhood obesity is one of the most consequential public health challenges of our era, functioning as a decisive determinant of lifelong risk for non-communicable diseases (NCDs) such as cardiovascular disease and type 2 diabetes (Juonala et al., 2011; Baker et al., 2007). Landmark longitudinal cohort evidence demonstrates that individuals who remain obese continuously from childhood into adulthood exhibit sharply elevated relative risks of type 2 diabetes, hypertension, and carotid atherosclerosis compared with those who maintain normal weight (Juonala et al., 2011). Childhood overweight is itself a strong antecedent: across systematic-review evidence, overweight children carry a substantially increased relative risk of adult obesity that rises with age (Simmonds et al., 2016).

Crucially, however, this trajectory is not deterministic. Individuals who, despite childhood obesity, achieve remission to normal weight before adulthood attenuate these disease risks to levels statistically comparable with peers who were never obese (Juonala et al., 2011). An epidemiological study of over 62,000 boys further showed that overweight at age 7 conferred no excess diabetes risk provided that normal weight was regained before puberty (Bjerregaard et al., 2018). This reversibility is the foundation of a preventive paradigm: the goal is to shift the developmental trajectory before obesity becomes established.

The developmental roots of this risk are also informed by the Developmental Origins of Health and Disease (DOHaD) hypothesis, which holds that maternal nutritional imbalance during pregnancy can epigenetically program fetal metabolism, predisposing offspring to later adiposity (Barker, 1990; Wu et al., 2004; Voerman et al., 2019). DOHaD offers a useful rationale for why early life matters, but its programming effects are fixed largely before and around birth and are not the target of the present intervention. The locus of intervention here is instead the modifiable postnatal trajectory - the child’s and family’s ongoing diet and physical activity after birth - since childhood overweight is reversible when addressed early (Juonala et al., 2011; Bjerregaard et al., 2018).

### 1.3 The promise and delivery gap of lifestyle intervention

Lifestyle guidance is clinically effective, though the strongest trial evidence comes from the perinatal window rather than from postnatal family counseling of the kind evaluated here. A Cochrane systematic review of 65 trials (over 11,000 participants) confirmed that diet and/or exercise interventions during pregnancy reduce the risk of excessive gestational weight gain by approximately 20% and lower perinatal complications including macrosomia and cesarean delivery (Muktabhant et al., 2015). Whether comparably delivered guidance is similarly effective for mothers of children already born - the postnatal population addressed by the present study - is less thoroughly established in controlled trials, although the underlying behavior-change principles are not thought to be specific to a single life stage. In either setting, the challenge is not efficacy but delivery at scale. In routine practice, providing continuous, individualized dietary and physical-activity guidance is severely constrained: clinicians face very short consultation windows, limited nutrition-counseling training, and chronic understaffing, while families are reluctant to accept additional appointments and time burdens (Schulz et al., 2022). Bridging proven efficacy and limited real-world delivery requires a dynamic, non-face-to-face guidance system that functions within each family’s own living environment, without consuming scarce clinical resources - and that delivers the individualized, precision-preventive care described in Section 1.1.

### 1.4 Digital twins as the engine of precision prevention

A promising means to deliver such individualized prevention is the digital twin - a virtual representation that not only mirrors an individual’s current state but forecasts future states and feeds guidance back to them, in contrast to static records that merely accumulate data. Extending this predictive capacity to the early-childhood setting - simulating a child’s future overweight risk from routinely collected family and infant data - converts the abstract, temporally distant threat of childhood obesity into a concrete, visualizable, and, critically, modifiable outcome. By rendering how a child’s projected trajectory responds to the family’s present lifestyle choices, the digital twin targets exactly the modifiable postnatal behavior identified in Section 1.2. It is this transformation of distant, averaged risk into present-tense, individualized motivation - communicated through empathic dialogue - that the present study sets out to build and validate; Section 2 reviews the digital-twin concept and related technical foundations in detail.

## 2 Related work

### 2.1 Predictive models and digital twins across clinical domains

Before turning to childhood overweight specifically, it is useful to establish that predictive digital twins already have precedent across multiple areas of adult chronic disease, since this precedent both motivates and de-risks the approach adopted here. In cardiovascular medicine, Coorey et al. (2022) conducted a mapping review of the health digital twin concept, searching the peer-reviewed literature for cardiovascular applications; they found that most cardiovascular digital twin research remained at the proof-of-concept and model-validation stage, with numerical simulation models predominating over fully realized, continuously updating real-time systems, and they highlighted persistent implementation challenges, including limited large-scale clinical validation and unresolved ethical and regulatory questions around AI-derived decision tools (Coorey et al., 2022). This review is instructive because it shows that even in a data-rich, heavily funded specialty like cardiology, the digital twin concept remains an emerging translational field rather than a mature clinical tool.

A second precedent comes from oncology. Ștefănigă et al. (2024) conducted a scoping review of digital and virtual twins in cancer care and found that such twins had already been applied to diagnosing, treating, and monitoring patients with breast, lung, and gastrointestinal cancers, and to pain management, with twins combining imaging, histology, and multi-omics data allowing clinicians to simulate a tumor’s response to chemotherapy, radiation, or immunotherapy before committing to a real-world treatment plan (Ștefănigă et al., 2024). At the same time, the authors cautioned that the twinning solutions they reviewed frequently lacked fairness and external validation, calling for continued multidisciplinary development supported by evolving regulatory frameworks before such twins could be trusted in routine oncology practice.

Together, these two reviews illustrate a consistent pattern: digital twins are demonstrably useful for translating multidimensional patient data into individualized, forward-looking simulations, but the field as a whole remains early-stage, with validation, fairness, and regulatory questions still open even in well-resourced adult disease areas. Pediatric and perinatal applications lag further behind. Machine-learning approaches to childhood obesity prediction have begun to emerge, though most have focused on short- to mid-term outcomes or have relied on data obtained after early infancy (Gupta et al., 2022). A more directly usable precedent for the present work is the model developed within the Tohoku Medical Megabank Birth and Three-Generation (TMM BirThree) cohort, which built multivariable logistic-regression models predicting overweight status at multiple ages from early childhood through adolescence using only routine life-course health records available up to 18–23 months of age - overweight status at 18–23 months, child’s sex, maternal age, parity, maternal BMI, maternal smoking and drinking, birth weight, and BMI change from birth to 18–23 months (Shinoda et al., 2026). Discrimination was moderate to high for early and middle childhood (AUC 0.873 at 36–47 months; 0.772 at 6 years) and modest for adolescence (AUC 0.720 and 0.692 at 11 and 14 years) (Shinoda et al., 2026). Because these predictors are drawn from routinely collected records, the model is scalable and directly translatable into existing child-health surveillance, and the present study adopts this published model rather than constructing a new one.

### 2.2 From digital shadows to predictive digital twins for health

A predictive equation alone is only a digital shadow until it is embedded in a system that acts back upon the patient. Three related but distinct concepts are often conflated here and are worth separating. A digital model is a static virtual representation with no automatic data exchange. A digital shadow adds a one-way, automatic data flow from the physical entity to its virtual representation - conventional electronic health records and most self-tracking applications fall here, faithfully accumulating data but unable to act back upon the patient. A digital twin completes the triad with a bidirectional, real-time information flow (the digital thread) connecting the physical entity, its virtual replica, and predictive simulation, so the model not only mirrors the patient but forecasts future states and feeds individualized guidance back to them (Vallée, 2023). A scoping review organized this field around properties for personalized and preventive care - personalization, interconnection, interactivity, beneficence, and impact - and underscored the shift from static records toward dynamic simulation (Katsoulakis et al., 2024). Implementations such as the Twin Health diabetes platform exemplify this potential: a digital-twin system integrating multidimensional data (continuous glucose monitoring, diet, step counts) dynamically predicted glycemic response and, in a one-year randomized controlled trial, achieved marked reductions in HbA1c, high remission rates, and improved cardiovascular risk factors (Shamanna et al., 2024). These systems establish that predictive simulation, embedded in an actionable loop, is feasible. What they generally lack is an interface capable of translating a predicted trajectory into empathic, individualized, and persuasive communication that a non-expert family member will act upon - the role we assign to a large language model.

### 2.3 Open-source medical LLMs: the limits of off-the-shelf models, RAG, and the case for SFT

The central technical question is how to make an open-source LLM deliver nutrition guidance that is reliably accurate and safe. Recent evidence makes the difficulty concrete. Parameswaran et al. (2025) evaluated off-the-shelf and retrieval-augmented models on guideline-adherent nutrition questions for cardiovascular-disease prevention and found that the off-the-shelf Llama 3-70B produced inappropriate responses on 40% of questions (12 of 30), the highest harm rate among the models tested. Grounding the same model in a curated guideline knowledge bank via retrieval-augmented generation (RAG) reduced inappropriate responses to 3% (1 of 30) with no detected harm (Parameswaran et al., 2025). RAG is therefore a powerful accuracy lever.

Crucially, however, that same study documents RAG’s principal weakness: instability. Even the RAG-enhanced model gave materially different answers to identical questions across repeated attempts - for example, varying its stated sodium target between attempts - and the authors note that RAG performance depends on external retrieval sources and is susceptible to model drift and version opacity, requiring ongoing validation (Parameswaran et al., 2025). Because RAG must perform a retrieval step against an external, often network-hosted, knowledge source at inference time, its outputs are contingent on the availability, versioning, and latency of that retrieval pipeline; in a child-health service expected to run continuously and within institutional networks, this network dependence is an operational and reliability liability.

This motivates supervised fine-tuning (SFT) as a complementary route: rather than retrieving knowledge at inference time, SFT internalizes domain knowledge into the model’s own weights, yielding a self-contained model that runs deterministically and locally without a live retrieval dependency. A substantial body of work shows that high-quality, instruction-formatted SFT reliably improves accuracy on specialized medical tasks: PMC-LLaMA adapted a general-purpose Llama model to medicine through data-centric knowledge injection - integrating 4.8 million biomedical papers and 30,000 medical textbooks - followed by comprehensive instruction fine-tuning on medical QA, reasoning rationale, and conversational dialogue, yielding substantial gains on medical QA benchmarks (Wu et al., 2024), and BioInstruct improved biomedical question answering, information extraction, and generation via LoRA-based tuning (Tran et al., 2024). The decisive lesson is that performance is driven by data quality, task alignment, and label design rather than raw volume (Griot et al., 2024; Wang et al., 2024). Parameter-efficient methods make this practical and privacy-preserving: LoRA freezes the base weights and trains low-rank matrices in the attention layers (Hu et al., 2022), and QLoRA-based tuning of Llama 3.1 has matched or exceeded full fine-tuning and BERT baselines (Liu et al., 2025).

This also reinforces the choice of an open-source foundation. Proprietary, closed API services (e.g., commercial GPT or Gemini offerings) expose a clinical system to vendor lock-in - pricing, availability, versioning, and data-handling terms are vendor-controlled and may change or be deprecated, and records must leave the institution to be processed. Building on Llama 3 (Grattafiori et al., 2024) allows local hosting, version-pinning, and tuning on sensitive child-health data without third-party transmission. The present work therefore adopts the same base model and the same registered-dietitian-anchored evaluation paradigm as Parameswaran et al. (2025), but replaces the network-dependent RAG layer with a reproducible SFT procedure grounded in the national food composition database - establishing a vendor-independent, self-contained methodology for specializing open-source models to the child-nutrition domain.

### 2.4 Evaluation of medical and nutrition-guidance LLMs

Evaluation scales for both general medical LLMs and, in particular, nutrition-guidance LLMs remain notably immature. Most available instruments are narrow in scope, developed for a single language or health system, cover only a fraction of real-world clinical scenarios, and have rarely been validated independently across multiple models. This scarcity of validated, domain-appropriate benchmarks is itself an important constraint on the field, and it directly shapes how any newly fine-tuned model - including the one developed here - can be responsibly evaluated.

For general clinical safety and factual accuracy in Japanese-language medicine, instruments exist that assess broad medical knowledge; Kanzawa et al. (2024), for example, showed that a fine-tuned Japanese LLM could reach expert-level accuracy in classifying structured clinical reports, demonstrating that high-accuracy Japanese medical LLMs are achievable in principle. Such instruments, however, are built to assess general medical knowledge rather than the specific competencies of dietetic and nutritional counseling; a nutrition response that is medically safe in a broad sense can still be nutritionally inappropriate - wrong in its sodium target, portion guidance, or interpretation of a food-composition value - in ways a general medical benchmark would not detect.

A nutrition-specific standard does exist, but not in a form directly usable here. Azimi et al. (2025) benchmarked three leading proprietary LLMs against the Registered Dietitian (RD) exam administered by the U. S. Commission on Dietetic Registration, using 1,050 multiple-choice questions spanning four domains of dietetic competency, and found that all three models exceeded the human passing threshold, scoring above 88%, although accuracy and consistency varied considerably by prompting technique and question domain (Azimi et al., 2025). This work demonstrates that a rigorous, nutrition-specific benchmark is achievable, but it is built around a U. S. professional credentialing exam and has no Japanese-language or Japan-relevant equivalent.

This gap - a Japanese, child-nutrition-specific accuracy benchmark simply does not yet exist - is precisely why the present study, like Parameswaran et al. (2025), evaluates response appropriateness through direct expert judgment by registered dietitians rather than through an omnibus benchmark score. Constructing such a benchmark, ideally aligned with the national food composition database and Japanese dietary reference intakes, is an important direction for future work, discussed further in Section 5.7.

### 2.5 Empathic dialogue, persuasive technology, and the behavior-change gap

Accuracy alone does not change behavior. Ayers et al. (2023) compared physician and AI chatbot responses to real patient questions drawn from a public social-media health forum and found that the chatbot’s responses were rated of higher quality and substantially more empathetic than physicians’ responses, even though the replies were also considerably longer (Ayers et al., 2023). This finding matters here because it shows, in a real clinical-question setting rather than a laboratory task, that a well-prompted LLM can consistently produce warmer, more supportive responses than time-pressured human clinicians - the same empathic capacity our system asks the fine-tuned LLM to exercise.

Such empathy can be designed for rather than left to chance. Michie et al. (2011) introduced the Behavior Change Wheel and its COM-B model, framing any behavior as the product of Capability, Opportunity, and Motivation; we adopt this as the organizing logic of our dialogue design, with the digital twin’s predicted trajectory targeting motivation, the chat interface’s low-effort logging targeting opportunity, and the LLM’s guidance targeting capability. This is complemented by the persuasive-technology principle that self-monitoring and immediate, personalized feedback reliably trigger durable habit change (Fogg, 2003).

The closest behavioral analogue to our own design comes from Mönninghoff et al. (2022), whose FutureMe randomized controlled trial showed that visualizing an age-progressed avatar of one’s own future self significantly increased daily step counts and shifted food purchases toward more healthful choices, an effect attributed to overcoming the present bias that ordinarily discounts distant health consequences. Where FutureMe visualizes a person’s own aged self, our digital twin visualizes a woman’s future child, applying the same present-bias-correcting logic to an arguably even more motivating target.

At the same time, the evidence carries an important caution. Entenberg et al. (2023) found that a brief AI chatbot micro-intervention for parents achieved meaningful engagement and short-term learning gains, but that evidence for durable behavioral change from a short, single-format chat interaction was limited. This draws a consistent boundary: empathic, personalized delivery reliably improves engagement and short-term motivation, but a short chat interaction alone does not reliably produce durable behavior change unless grounded in something more substantial than dialogue quality - precisely the gap the digital twin is designed to fill.

### 2.6 Positioning of the present work

Looking further ahead, even once technology capable of highly accurate prediction of disease risk and health-habit trajectories is fully established, an important research question will remain open: whether presenting that prediction through a visual application such as a digital twin, and explaining it interactively through conversational dialogue, actually produces behavior change. Linking accurate prediction, visual embodiment, and conversational persuasion into a single causal chain has not yet been established as a mature area of research.

Our own group has been building toward this question in a stepwise fashion. In an earlier pilot study, we combined wearable-device activity data with a metaverse environment in which participants’ digital-twin avatars visualized both current health metrics and a predicted future body composition; even without any conversational or chat function, this passive visualization was associated with a significant increase in daily step counts and high subjective satisfaction among older adults (Nakamura et al., 2025). We subsequently extended this line of work with a tablet-based virtual community application in which shared avatars, driven by wearable data, were combined with lightweight social features in older adult care facilities; this pilot feasibility study found that avatar-sharing and social feedback sustained improvements in step count and medication adherence considerably better than reminder notifications alone (Nakamura et al., 2026).

These two studies establish that visual, avatar-based digital twins can motivate behavior change, and that light social interaction can help sustain it - but neither incorporated a conversational agent capable of explaining an individualized prediction in natural language. The present study is the next step in this trajectory: to our knowledge, it is a first attempt to combine a cohort-validated predictive digital twin with an empathic, supervised-fine-tuned nutrition-guidance LLM used as a conversational agent, so that the predicted trajectory is not only visualized but actively explained and discussed with the person it concerns. Whether this combination of visual prediction and interactive, dialogue-based explanation produces behavior change more effectively than either element alone remains an open empirical question. The present, single-arm study does not itself isolate that comparative effect (Section 5.6); rather, it establishes the feasibility and acceptability of the combined system as a necessary first step toward answering it.

To align the study’s aims with the evaluation components reported below, the specific objectives of this study were: (1) to assess the appropriateness of the fine-tuned nutrition-guidance LLM as judged by registered dietitians; (2) to assess the acceptability of the digital-twin visualization among mothers of young children (defined in this study as women with a child under 14 years of age); (3) to evaluate the acceptability of the chat-based consultation among mothers of young children; and (4) to identify the relationship between AI response characteristics and behavioral intention among mothers of young children.

## 3 Methods

### 3.1 Study overview: an integrated intervention with multiple evaluation components

To ensure system reliability and evaluate acceptability and behavioral intention, a single integrated intervention - combining a fine-tuned nutrition-guidance LLM, a digital twin, and a chat-based consultation - was developed and evaluated through multiple complementary evaluation components rather than sequential trial phases. The first component constructed and evaluated a domain-specialized, open-source Japanese nutrition-guidance LLM via supervised fine-tuning. The second component built a digital twin using a published, cohort-validated childhood-overweight prediction model (Shinoda et al., 2026) and tested whether visualizing a child’s future overweight risk elicits behavioral intention toward change in 121 women with a child under 14 years of age. The third component deployed the integrated system in a single chat-based test with the same 121 participants, evaluating whether one interaction was associated with behavioral intention toward change. The primary outcome was self-reported behavioral intention toward change following the digital-twin visualization and chat consultation; secondary outcomes included perceived value of the consultation, willingness to continue, willingness to pay for an extended consultation, willingness to use tracking or disease-risk-feedback applications, and thematic categories derived from chat content. The studies involving human participants were approved by the Ethics Committee of Gunma University Graduate School of Medicine. The studies were conducted in accordance with local legislation and institutional requirements. The participants provided written informed consent to participate in this study. Data were collected between October 2025 and March 2026.

In terms of design, this was a single-arm, single-session feasibility and acceptability study; no control or comparison arm was included. The complete intervention was delivered as a web-based application, which each participant accessed on an 11-inch iPad (Apple Inc.) provided by the investigator during a single on-site session. Within that session, each participant first viewed the digital-twin visualization and completed its three-item questionnaire, and then completed the chat-based test (up to five minutes of interaction) followed by the six-item chat-consultation questionnaire; the digital-twin visualization, the chat consultation, and all questionnaires were therefore delivered on the same web-based platform and completed consecutively, with the full session lasting approximately 15–20 min per participant. Eligibility required being a woman with a child under 14 years of age who was able to provide written informed consent; individuals unable to consent or without an eligible child were excluded. All 121 participants who consented and began the session completed both the digital-twin and chat-based components and are included in the analysis, with no drop-outs and no missing questionnaire responses (no missing-data handling was therefore required). The numbers of individuals approached or declining participation prior to informed consent were not systematically recorded, so the recruitment flow is reported from consent onward.

The 121 participants were women each with a child under 14 years of age, recruited from the public in Gunma Prefecture, Japan, among those who provided informed consent. The intervention was designed as a universal, primordial-prevention tool intended for the general population of mothers of young children, rather than as a treatment for children who are already overweight; accordingly, participants were recruited from the community without screening on maternal or child body-mass index, and the resulting near-normal mean maternal BMI (21.37 kg/m^2^) is consistent with, rather than contrary to, this population-level preventive aim. Children’s body-mass index was not measured or recorded in this study, so the sample is best characterized as a general community population rather than a clinically selected high-risk group. Their mean maternal age was 29.3 ± 5.6 years and mean maternal BMI was 21.37 ± 4.44 kg/m^2^. Participant background characteristics are summarized in Table 1. Child-level and pregnancy-related values required to generate each participant’s digital-twin visualization (child’s sex, birth weight, and related handbook records; Section 3.3) were entered transiently during the session but were not retained as structured background data, and so are not reported in Table 1.

**Table 1:** Participant background characteristics (*N* = 121).

Characteristic	Value
Maternal age, years (mean ± SD)	29.3 ± 5.6
Maternal BMI, kg/m^2^ (mean ± SD)	21.37 ± 4.44
Recruitment method	Public recruitment; participants who provided informed consent
Exclusion criteria	Individuals unable to provide informed consent; those without an eligible child

### 3.2 Construction and evaluation of the nutrition-guidance LLM

For the reasons developed in Section 2.3, we built on the open-source Llama 3-70B model (Grattafiori et al., 2024) rather than a closed commercial API, enabling local hosting, version-pinning, and fine-tuning on sensitive child-health data without third-party transmission. Our experimental procedure deliberately mirrors that of Parameswaran et al. (2025) - the same base model (Llama 3-70B) and the same registered-dietitian-anchored evaluation of response appropriateness - but substitutes their network-dependent RAG layer with supervised fine-tuning on an authoritative nutritional knowledge source, so that the resulting model is self-contained and deterministic at inference time. As discussed in Section 2.4, no Japanese nutrition-specific benchmark currently exists to certify such a model directly, which is why direct expert evaluation, rather than an omnibus benchmark score, was adopted as the primary accuracy measure.

The authoritative knowledge source underlying the fine-tuning data was the Standard Tables of Food Composition in Japan 2020 (Eighth Revised Edition) together with its 2023 amendment (Ministry of Education, Culture, Sports, Science and Technology (MEXT), Science and Technology Policy Bureau, Subdivision on Resources, 2020, 2023), which together list 2,481 food items organized into eighteen food groups spanning cereals, potatoes and starches, sugars, pulses, seeds and nuts, vegetables, fruits, mushrooms, algae, fish and shellfish, meat, eggs, milk and dairy products, fats and oils, confectionery, beverages, seasonings and spices, and prepared dishes. Each food item in this table is cross-referenced to supplementary amino-acid, fatty-acid, and carbohydrate composition tables, so that a single entry carries not only macronutrient values but also its amino-acid profile and fatty-acid breakdown. This structure was used directly in constructing the instruction data: guidance concerning protein quality could draw on the amino-acid sub-table for a recommended food, and guidance concerning fat quality could draw on the corresponding fatty-acid sub-table, rather than relying on the model’s own, potentially hallucinated, generalizations about a food’s composition.

Each training sample used an instruction–input–output format. Following the data-quality-first principle established in the instruction-tuning literature (Griot et al., 2024; Wu et al., 2024), outputs were not limited to numeric verdicts; the expert decision process of registered dietitians and obstetricians was explicitly annotated as an accompanying rationale, so the model learns the chain of clinical reasoning rather than isolated answers. A representative sample comprises an instruction to generate a nutritional assessment and supportive feedback from a participant’s measured body-composition profile together with her life-stage context, an input consisting of the participant’s height, weight, body fat mass, and BMI mapped onto the corresponding food-group and sub-table entries, an output that classifies the participant’s status against the reference range and translates that classification into concrete, food-group-anchored guidance expressed in familiar portions and everyday foods rather than abstract nutrient targets, and a rationale annotation that ties each recommendation back to the participant’s specific measured values and to the relevant food-composition entries. For example, a profile falling below the reference range is paired with guidance that emphasizes energy-dense, easily digested foods, a pattern of three main meals supplemented by energy-dense snacks, and generous use of quality fats such as olive oil, nuts, and oily fish to raise caloric intake without increasing meal volume, whereas a profile falling above the reference range is paired with guidance that emphasizes fiber-rich, minimally processed staples, a vegetable-first eating order, and snack substitution toward yogurt, nuts, or fruit rather than confectionery, in both cases with the specific recommended foods and portions drawn from the food-composition table’s groupings rather than generated freely by the model.

Model fine-tuning and inference were conducted locally on a workstation equipped with an Intel Xeon Silver 4,510 CPU, 128 GB DDR5 ECC memory, a 1.92 TB SSD, and one NVIDIA RTX 6000 Ada GPU running Ubuntu, consistent with the study’s emphasis on locally controllable, vendor-independent deployment (Section 2.3).

Domain knowledge was injected via Low-Rank Adaptation, implemented as quantized LoRA (QLoRA) with 4-bit NF4 quantization of the base model (Hu et al., 2022). This configuration was chosen because parameter-efficient, quantized fine-tuning matches or exceeds full-parameter fine-tuning on specialized clinical tasks while enabling secure, memory-efficient local tuning on a single workstation GPU (Liu et al., 2025). The instruction dataset comprised 2,481 instruction–input–output samples, one for each food item in the national food composition database, and was partitioned under a 90:10 split into 2,233 training and 248 validation samples. With an effective batch size of 16 over three epochs, this corresponded to 420 optimization steps. Table 2 lists the principal fine-tuning hyperparameters. A system prompt prohibited definitive diagnosis and discouraged direction toward unsupervised medical actions, serving as a safety guardrail.

**Table 2:** Principal hyperparameters used for QLoRA/LoRA fine-tuning of the base Llama 3-70B model.

Parameter	Value
Base model	Llama 3-70B
Fine-tuning method	QLoRA / LoRA
Quantization	4-bit NF4
LoRA rank	16
LoRA alpha	32
LoRA dropout	0.05
Target modules	q_proj, k_proj, v_proj, o_proj
Learning rate	2e-4
Optimizer	Paged AdamW 8-bit
Batch size	1
Gradient accumulation steps	16
Effective batch size	16
Epochs	3
Max sequence length	2,048 tokens
Warmup ratio	0.03
Weight decay	0.01
LR scheduler	Cosine
Precision	bf16 if available, otherwise fp16
Gradient checkpointing	Enabled
Evaluation strategy	Every epoch
Early stopping	Patience = 2
Random seed	42

To evaluate the effect of SFT, we adopted the appropriateness-evaluation paradigm of Parameswaran et al. (2025): their 30-item cardiovascular-nutrition question set was posed to the model, organized across eight major categories - fundamentals of a heart-healthy diet, cooking and food selection, salt and sodium intake, the three macronutrients, individual food groups, beverages, popular and specialized diets, and individualization and safety. Example items included questions about sodium restriction, appropriate daily protein intake, higher-protein snack options, beverage choices, and safe dietary guidance for weight management; the full 30-item set, its category assignments, and the per-item ratings are provided in Supplementary Table S1. Responses were independently judged appropriate or inappropriate by three registered dietitians (denoted A, B, and C), and the off-the-shelf Llama 3-70B was compared against the SFT-tuned model; the results of this evaluation are reported in Section 4.1.

### 3.3 Digital twin construction and self-assessment evaluation

The digital twin’s predictive engine was not newly constructed; it adopts the published, cohort-validated logistic-regression model developed in the Tohoku Medical Megabank Birth and Three-Generation (TMM BirThree) cohort (Shinoda et al., 2026). That model predicts the probability of a child being overweight at 36–47 months, 6 years, 11 years, and 14 years of age using routine life-course health records available up to 18–23 months of age - overweight status at 18–23 months, child’s sex, maternal age at delivery, parity, maternal BMI, maternal smoking and drinking at pregnancy recognition, birth weight, and BMI change from birth to 18–23 months - with reported discrimination of AUC 0.873 (36–47 months), 0.772 (6 years), 0.720 (11 years), and 0.692 (14 years) (Shinoda et al., 2026). Because these inputs are drawn from records that are routinely retained for every child in Japan’s maternal and child health handbook system, they remain available regardless of a child’s current age, and we use these published regression equations directly as the prediction layer of the digital twin. Predictor variables required by the Shinoda et al. model–including the child’s sex, birth weight, and overweight status at 18–23 months–were obtained from participant-reported maternal and child health handbook records; when an exact value was unavailable, participants were asked to enter the closest available record, and no imputation was performed. These values were entered transiently by each participant solely to generate her own visualization and were not retained as structured background data for cohort-level reporting; they therefore do not appear in the participant-characteristics summary in Section 3.1. Where individual records were incomplete, the corresponding visualization should be understood as a simulation-based illustration rather than a fully individualized prediction.

The digital twin renders the predicted trajectory visually and dynamically: rather than issuing a uniform notice, it shows how the child’s projected overweight risk shifts in response to modifiable family-level inputs (e.g., dietary balance and physical-activity patterns), giving participants concrete outcome predictability over the modifiable postnatal course. The 121 participants were shown this simulated future risk for their child, and we evaluated whether the visualization elicited a behavioral intention toward change. The digital-twin visualization and the chat-based test were administered within the same session, immediately after written informed consent was obtained, so that the two evaluation components occurred consecutively for each participant rather than at separate visits.

**Figure 1:**
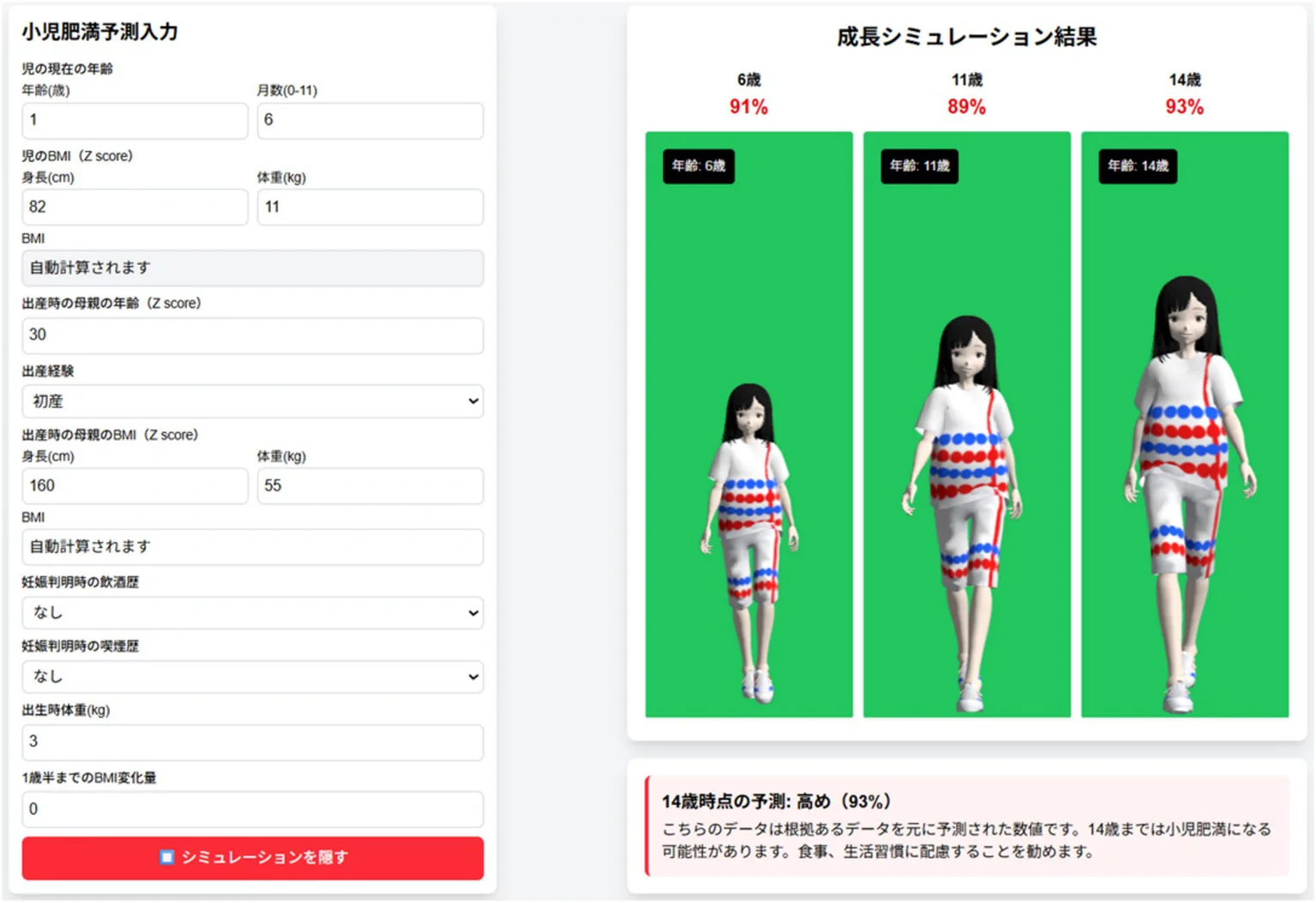
The digital-twin screen used in this evaluation. The left panel shows the child and maternal variables entered for the prediction model. The right panel displays simulated child avatars and predicted overweight risks at 6, 11, and 14 years. In the example shown, the predicted risks are 91%, 89%, and 93%, respectively.

Following the visualization, self-reported outcomes were captured using a three-item digital-twin questionnaire administered on a five-point scale (1 = strongly disagree, 5 = strongly agree): the clarity of the application’s user interface, whether the visualization prompted a behavioral intention toward change, and willingness to continue attending future health consultations. This instrument was developed originally for this study and has not undergone formal psychometric validation (Figure 1).

### 3.4 Chat-based test and self-assessment evaluation

The same 121 participants each completed a single chat-based test via a dedicated web application, designed to assess whether one interaction with the system was associated with behavioral intention toward change. Through the chat interface, each participant entered dietary, step-count, and body-composition information and received individualized feedback grounded in the digital twin’s predicted risk trajectory. The feedback dialogue implemented the empathic clinical-response logic validated by Ayers et al. (2023) - generating warm, supportive expressions rather than terse factual statements - integrated with the COM-B model and Behavior Change Wheel to strengthen capability, opportunity, and motivation (Michie et al., 2011). The COM-B model and the Behavior Change Wheel informed the design of the chat-based feedback component specifically - together with the motivational framing of the digital-twin visualization - rather than the construction of the nutrition-guidance LLM, whose fine-tuning targeted factual appropriateness. Concretely, the intervention components were mapped to the COM-B domains as follows. Capability was addressed by translating each participant’s profile into concrete, food-group-anchored guidance expressed in familiar portions and everyday foods, so that recommendations were understandable and enactable. Opportunity was addressed through the low-effort logging interface and by framing changes as small, low-burden adjustments that fit existing family routines - for example, a vegetable-first eating order, or substituting yogurt, nuts, or fruit for confectionery - rather than as demands for new resources. Motivation was addressed jointly by the digital twin’s visualization of a modifiable, individualized future-risk trajectory for the child and by the LLM’s empathic, encouraging delivery, which reframed the predicted trajectory as changeable rather than fixed. In practice, each participant’s measured body-composition profile was classified against the reference range and translated into the same food-group-anchored guidance that structured the fine-tuning data (Section 3.2) - energy-dense recommendations for profiles below the reference range and fiber-rich, vegetable-first recommendations for profiles above it - but delivered here with encouragement and concrete, achievable steps rather than as a directive to restrict.

**Figure 2:**
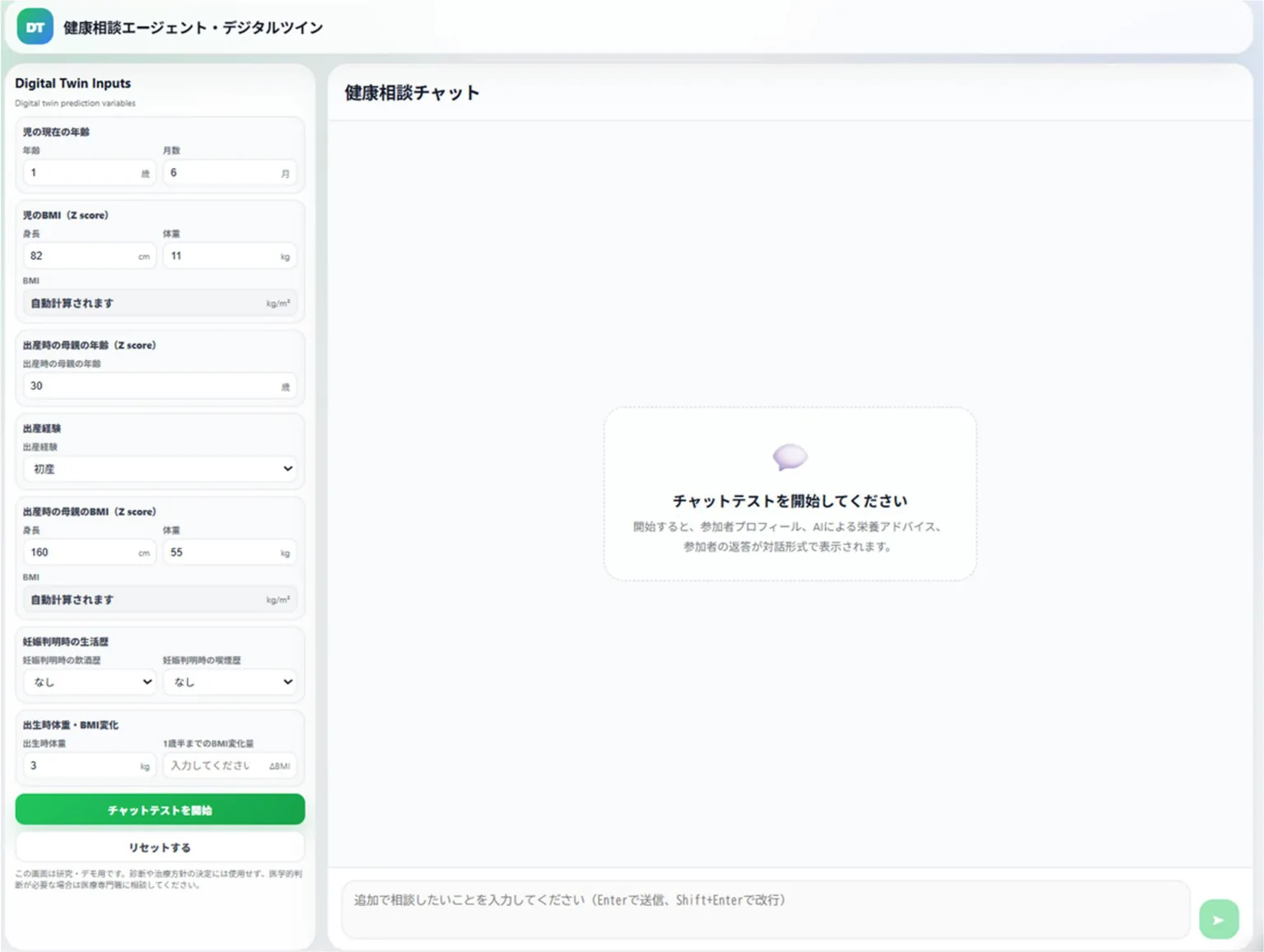
The chat-interface screen used in this evaluation. The left panel shows child and maternal health-information fields used by the system. The right panel shows the chat area before initiation of the consultation, with a prompt to start the chat test. No personally identifying information is displayed.

Following the single interaction, acceptability and perceived behavioral impact were measured using a six-item chat-consultation questionnaire administered on a five-point scale (1 = strongly disagree/not at all willing, 5 = strongly agree/very willing), distinct from the three-item digital-twin questionnaire described in Section 3.3. The six items covered the perceived value of the consultation, whether it prompted a behavioral intention toward change, willingness to continue attending future consultations, willingness to pay for an extended, thirty-minute consultation, willingness to use an application for ongoing body-composition tracking and consultation, and willingness to use an application providing disease-risk feedback from body-composition data. The full item wording is reported alongside the corresponding results in Section 4.3. As with the digital-twin questionnaire, this instrument was developed originally for this study and has not undergone formal psychometric validation (Figure 2).

### 3.5 Statistical analysis

Descriptive statistics summarized participant characteristics and survey outcomes, including means and standard deviations for each questionnaire item, computed using R. No missing-data handling (e.g., imputation) was applied. Internal consistency of the two three-item self-assessment scales was evaluated with Cronbach’s *α*, which was 0.82 for the digital-twin scale (Q1–Q3) and 0.91 for the first three items of the six-item chat-consultation questionnaire (Q1–Q3; Section 3.4), indicating good to excellent reliability. Free-text content entered during the chat-based test was additionally examined using thematic content analysis to identify recurring participant themes, independent of the structured questionnaire ratings; this coding was performed without formal inter-rater reliability assessment.

For the accuracy evaluation of the fine-tuned model (Section 4.1), each response was independently rated by three registered dietitians; their per-item ratings are provided in Supplementary Table S1, and inter-rater agreement was quantified with Fleiss’ kappa computed from these per-item ratings (Section 4.1).

For the exploratory analysis reported in Section 4.4, AI-generated responses were additionally coded for the presence or absence of four characteristics: empathic/supportive expressions, protein-related guidance, exercise recommendations, and vegetable/fiber-related guidance. Behavioral-intention scores were compared between participants who did and did not receive each response characteristic using the Mann–Whitney U test, and effect sizes were quantified with Cliff’s delta (*δ*), with |δ| below 0.15, 0.33, and 0.47 taken to indicate a negligible, small, and medium effect, respectively. These analyses were exploratory and were not adjusted for multiple comparisons.

## 4 Results

**Figure 3:**
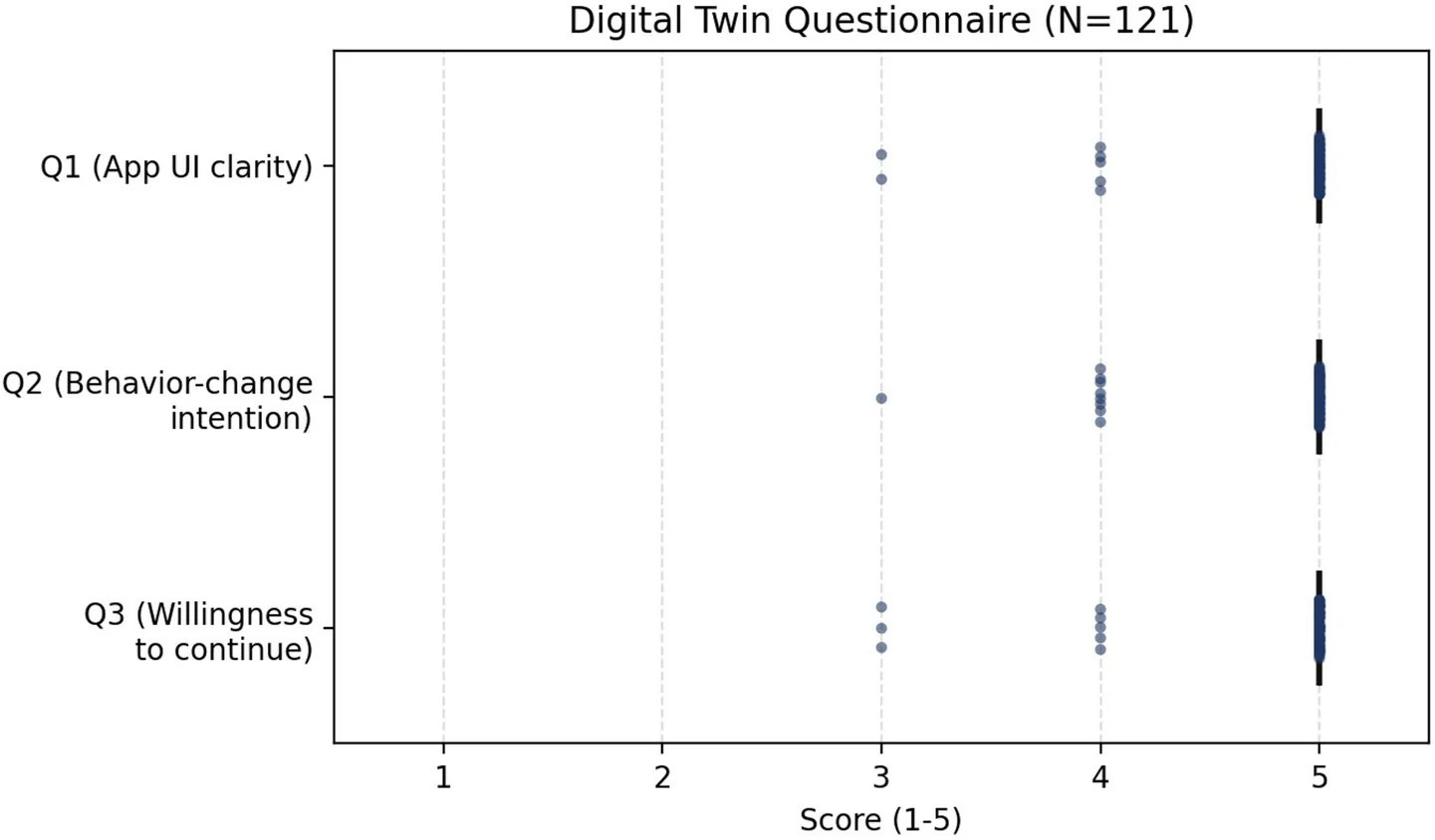
Distribution of responses to the digital-twin self-assessment questionnaire (5-point scale; *N* = 121), shown as box plots overlaid with beeswarm plots of individual responses. Box: Interquartile range; Horizontal line: Median; Whiskers: Range excluding outliers; Points: Individual participant responses (jittered horizontally for visibility).

**Figure 4:**
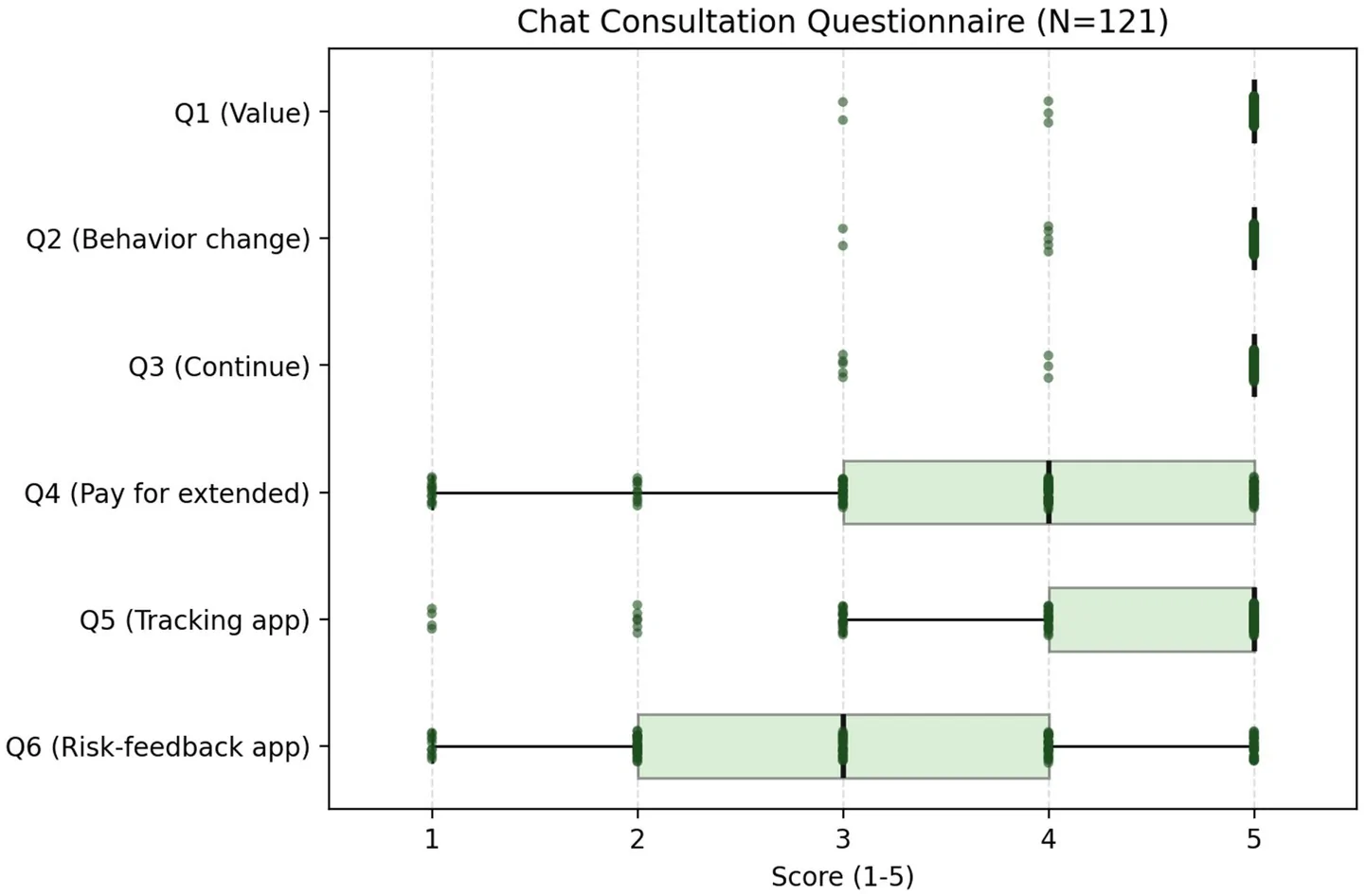
Distribution of responses to the chat-consultation self-assessment questionnaire (5-point scale; *N* = 121), shown as box plots overlaid with beeswarm plots of individual responses. Box: interquartile range; horizontal line: median; whiskers: range excluding outliers; points: individual participant responses (jittered horizontally for visibility).

This section reports the principal findings, organized by evaluation component: Section 4.1 reports the nutrition-guidance-LLM accuracy evaluation (Table 3), Section 4.2 reports the digital-twin self-assessment questionnaire (Figure 3), and Section 4.3 reports the text-chat self-assessment questionnaire (Figure 4) together with a thematic analysis of chat content (Table 4).

**Table 3:** Evaluation of response appropriateness on the 30-item question set, by model condition and by each of three registered dietitians (A, B, C), with the three-rater average.

Model condition	Dietitian	Appropriate	Inappropriate	Reference comparison
Off-the-shelf Llama 3-70B	A	19/30 (63.3%)	11/30 (36.7%)	
Off-the-shelf Llama 3-70B	B	17/30 (56.7%)	13/30 (43.3%)	
Off-the-shelf Llama 3-70B	C	19/30 (63.3%)	11/30 (36.7%)	
Off-the-shelf Llama 3-70B	Average	18/30 (60.0%)	12/30 (40.0%)	12/30 (40.0%) (Parameswaran et al., 2025)
Llama 3-70B + SFT (food composition DB)	A	27/30 (90.0%)	3/30 (10.0%)	
Llama 3-70B + SFT (food composition DB)	B	26/30 (86.7%)	4/30 (13.3%)	
Llama 3-70B + SFT (food composition DB)	C	28/30 (93.3%)	2/30 (6.7%)	
Llama 3-70B + SFT (food composition DB)	Average	27/30 (90.0%)	3/30 (10.0%)	-

SFT denotes supervised fine-tuning on the national food composition database. Individual rater appropriate-response counts ranged from 17 to 19 for the off-the-shelf model and from 26 to 28 for the SFT model; per-item ratings are provided in Supplementary Table S1. Three-rater averages (18.3/30 and 11.7/30 for the off-the-shelf model; 27.0/30 and 3.0/30 for the SFT model) are rounded to the nearest whole response for readability.

**Table 4:** Thematic distribution of participant chat content during the single chat-based test (*N* = 121; themes are not mutually exclusive).

Rank	Theme	Participants	Illustrative content
1	Feasibility: starting gradually, without excessive burden	112 (93%)	"Where I can," "without strain," "little by little" - strong response to perceived feasibility
2	Weight management: maintenance, gain, or loss	91 (75%)	Concern about, or interest in maintaining, increasing, or decreasing body weight
3	Reassurance and psychological acceptance	77 (64%)	Frequent expressions of relief, indicating that the guidance reduced anxiety
4	Snacking, meal frequency, and eating-style adjustments	67 (55%)	Attention to concrete, easily implemented practical adjustments
5	Attention to protein intake	58 (48%)	References to protein and to meat, fish, eggs, and soy products

### 4.1 Accuracy of the fine-tuned nutrition-guidance LLM

Following the appropriateness-evaluation paradigm of Parameswaran et al. (2025), we reproduced their evaluation design using the same base model (Llama 3-70B) and the same registered-dietitian-anchored judgment of response appropriateness. The evaluation set comprised 30 nutrition questions organized into eight categories spanning the fundamentals of a heart-healthy diet, cooking and food selection, salt and sodium intake, the three macronutrients, individual food groups, beverages, popular and specialized diets, and individualization and safety (Section 3.2). To reduce the influence of any single evaluator, appropriateness was judged independently by three registered dietitians, denoted A, B, and C, and their ratings were averaged. Agreement among the three raters was high: individual appropriate-response counts ranged from 17 to 19 for the off-the-shelf model and from 26 to 28 for the SFT model, and item-level agreement was substantial to almost perfect (Fleiss’ kappa = 0.86 and 0.75, respectively; Supplementary Table S1).

Supervised fine-tuning on the national food composition database substantially improved the appropriateness of the model’s responses to these 30 questions relative to the off-the-shelf baseline. For the off-the-shelf Llama 3-70B, the three raters (A, B, C) judged 11, 13, and 11 responses inappropriate, respectively (mean 11.7 of 30, 39.0%) consistent with the rate reported for the same base model and question count by Parameswaran et al. (2025). After fine-tuning, the three raters judged 27, 26, and 28 responses appropriate, respectively (mean 27 of 30, 90.0%). The per-rater counts are shown in Table 3.

### 4.2 Digital twin self-assessment questionnaire

Following the digital-twin visualization, participants completed the three-item self-assessment questionnaire described in Section 3.3. Across the 121 participants, all three items were rated highly and with limited spread: the clarity of the interface had a mean of 4.93 (SD = 0.32, median 5), the behavioral-intention item had a mean of 4.92 (SD = 0.31, median 5), and willingness to continue had a mean of 4.91 (SD = 0.37, median 5). Figure 3 shows the distribution of responses to each item as a box plot overlaid with a beeswarm plot of individual responses; for all three items, the interquartile range sat at the ceiling of the scale, with a small number of individual responses at 3 or 4, visible as the scattered points below the box, accounting for essentially all of the observed spread.

### 4.3 Text-chat self-assessment questionnaire and chat-content analysis

Following the single chat-based test, participants completed the six-item chat-consultation questionnaire described in Section 3.4: perceived value of the consultation (Q1), whether it prompted a behavioral intention toward change (Q2), willingness to continue attending future consultations (Q3), willingness to pay for an extended, thirty-minute consultation (Q4), willingness to use an application for ongoing body-composition tracking and consultation (Q5), and willingness to use an application providing disease-risk feedback from body-composition data (Q6). As with the digital-twin questionnaire, the items concerning the consultation itself were rated near the ceiling of the scale, with means (SD) of 4.94 (0.30) for Q1, 4.93 (0.32) for Q2, and 4.89 (0.42) for Q3. Items concerning a prospective standalone application were more differentiated: Q4 (pay for an extended consultation) had a mean of 3.60 (SD = 1.26), Q5 (tracking application) had a mean of 4.25 (SD = 1.09), and Q6 (disease-risk-feedback application) had the widest spread and the lowest central tendency, with a mean of 3.27 (SD = 1.30) and a median of 3. Figure 4 shows the distribution of all six items as a box plot overlaid with a beeswarm plot of individual responses; the widening interquartile ranges and denser spread of individual points for Q4 through Q6, relative to the ceiling-clustered points for Q1 through Q3, are visually apparent.

Beyond the structured questionnaire, each of the 121 participants completed a single text-based chat test of up to 5 minutes’ duration, and this chat content was analyzed separately from the questionnaire responses. Engagement was limited but consistent: participants entered a mean of 1.8 messages during the session (range 0–4 messages per participant); a count of 0 indicates that the participant received the initial individualized feedback generated from their entered profile data but did not enter additional free-text messages. A thematic analysis of the chat content identified five recurring themes, summarized in Table 4. The most frequent theme, present in 112 of 121 participants (93%), reflected a perception that the recommended changes could be started gradually and without excessive burden, with responses emphasizing feasibility in phrases such as “starting where I can” and “little by little.” The second most frequent theme, present in 91 participants (75%), concerned weight management - expressions of concern about current weight or explicit interest in weight maintenance, gain, or loss. A third theme, present in 77 participants (64%), reflected reassurance and psychological acceptance of the guidance, frequently expressed as relief that the recommended changes were less demanding than anticipated. A fourth theme, present in 67 participants (55%), concerned concrete, easily actionable adjustments to snacking, meal frequency, and eating style. A fifth theme, present in 58 participants (48%), reflected explicit attention to protein intake, often referencing the specific food groups - meat, fish, eggs, and soy products - highlighted in the system’s guidance.

### 4.4 Relationship between AI response characteristics and behavioral intention

To further explore factors associated with participants’ behavioral intention, an exploratory analysis was conducted to examine the relationship between characteristics of AI-generated responses and questionnaire outcomes.

AI-generated responses were categorized into four characteristics: empathic/supportive expressions, protein-related nutritional guidance, exercise recommendations, and vegetable/fiber-related guidance. For each characteristic, behavioral-intention scores were compared between participants who did and did not receive it. Participants receiving empathic/supportive responses (e.g., encouraging gradual lifestyle modification and reassuring messages) reported higher behavioral-intention scores than those who did not (mean 4.96 vs. 4.79). This difference did not reach statistical significance (Mann–Whitney U test, *p* = 0.105) and corresponded to a negligible effect size (Cliff’s *δ* = 0.09).

No statistically significant associations were observed for protein-related guidance (*p* = 0.744), exercise recommendations (*p* = 0.374), or vegetable/fiber-related guidance (*p* = 0.755). Because almost all participants received protein-related guidance (119 of 121), the corresponding comparison involved only two non-recipients. These findings are exploratory and were not adjusted for multiple comparisons (Table 5).

**Table 5:** Behavioral-intention scores by AI response characteristic, comparing participants who did and did not receive each characteristic (*N* = 121).

AI response characteristic	Received, *n*	Received, mean ± SD	Not received, *n*	Not received, mean	*p*
Empathic/supportive responses	97	4.96 ± 0.21	24	4.79	0.105
Protein-related guidance	119	4.92 ± 0.31	2	5.00	0.744
Exercise recommendations	109	4.92 ± 0.32	12	5.00	0.374
Vegetable/fiber guidance	93	4.92 ± 0.30	28	4.93	0.755

Comparisons are exploratory (Mann–Whitney U test, unadjusted). Behavioral intention corresponds to the chat questionnaire Q2 item (5-point scale). Received-group values are shown as mean ± SD and not-received-group values as the mean, both computed from the raw item-level data. Because the Q2 scores were strongly skewed toward the ceiling (114 of 121 participants scored 5), the not-received-group means are uniformly high. For the empathic/supportive comparison, the effect size was negligible (Cliff’s δ = 0.09). The non-recipient groups for the remaining characteristics ranged from 2 to 28 participants. For protein-related guidance, only two participants were classified as non-recipients.

## 5 Discussion

### 5.1 Principal findings

This study set out to build and validate a precision-preventive intervention that integrates a digital twin with an empathic, supervised-fine-tuned (SFT) open-source nutrition-guidance LLM, and to test whether such a system can move participants toward behavioral intention for change in the prevention of childhood obesity. Four principal findings emerged. First, supervised fine-tuning of an open-source model on the national food composition database substantially improved the appropriateness of nutrition responses: the off-the-shelf Llama 3-70B produced inappropriate responses on 12 of 30 questions (40.0%) - identical to the rate independently reported for the same base model and question count by Parameswaran et al. (2025) - whereas after fine-tuning, a mean of 27 of 30 responses (90.0%) were judged appropriate across three registered dietitians. Second, the digital-twin visualization was rated very highly by the 121 participants across all three self-assessment items (means of 4.91–4.93 of 5, each with a standard deviation below 0.4). Third, the chat consultation was similarly well received on the items concerning the consultation itself (means of 4.89–4.94), while items concerning a prospective standalone application showed more differentiated, lower, and more variable ratings (means of 3.27–4.25, standard deviations of 1.09–1.30). Fourth, thematic analysis of the chat content itself showed that the dominant participant response, present in 93% of participants, was a perception that the recommended changes were feasible and could be started gradually, alongside substantial engagement with weight management (75%), reassurance and reduced anxiety (64%), concrete eating-behavior adjustments (55%), and protein awareness (48%). Together, these findings indicate that accurate, locally controllable domain specialization and empathic, individualized communication can be combined in a single deployable system that participants experience as both valuable and feasible to act on, even as enthusiasm for a fully autonomous companion application remains more measured.

### 5.2 SFT as a complementary, operationally self-contained approach to retrieval-augmented generation

A central methodological contribution is the observation that SFT can reach the same general order of appropriateness attributed to retrieval-augmented generation (RAG), in an indirect comparison, while offering a more operationally self-contained profile. Parameswaran et al. (2025) showed that RAG reduced inappropriate nutrition responses from 12/30 (40.0%) to 1/30 (3.3%), an impressive gain, but documented that even the RAG-enhanced model produced inconsistent answers across repeated queries and depended on external retrieval sources susceptible to drift and version opacity. Our SFT approach reached a broadly comparable appropriateness level (27/30, 90.0% appropriate; 3/30, 10.0% inappropriate) - though its inappropriate-response rate remains roughly three times higher than the 3.3% reported for the RAG-enhanced model - by internalizing domain knowledge into the model’s weights rather than retrieving it at inference time. It should be stated explicitly that SFT and RAG were not directly compared on identical questions within our own evaluation; the RAG figures above are drawn entirely from Parameswaran et al. (2025), and the comparison offered here is therefore indirect, resting on the assumption that the two studies’ question sets and dietitian-rating procedures are sufficiently similar to be juxtaposed. The practical consequence of our approach is a more operationally self-contained model that runs locally within institutional networks, without a live network dependency - an important property for a child-health service expected to operate continuously and to handle sensitive data. This positions SFT not as a replacement for RAG but as a complementary approach that may be preferable where reliability, reproducibility, and data sovereignty are paramount; a direct, within-study head-to-head comparison remains an important next step.

### 5.3 The value of open-source models and vendor independence

The choice of an open-source foundation (Llama 3-70B) is not incidental but integral to the contribution. The vendor-lock-in and data-sovereignty liabilities of closed, proprietary API services (Section 2.3) are particularly consequential for a child-health service intended for sustained deployment, where continuity of service and patient privacy cannot be subordinated to a single vendor’s commercial decisions. By establishing a reproducible SFT methodology on an open-source model, the present work yields a system that can be hosted, version-pinned, and tuned on sensitive child-health data without third-party transmission. More broadly, it suggests that moderately sized open-source models, properly specialized, can achieve promising preliminary levels of response appropriateness in a limited expert-rated evaluation - lowering the barrier to locally governed medical AI.

### 5.4 From accurate facts to behavioral intention: the role of empathy and the digital twin

Accuracy alone does not build behavioral intention, and our results speak to the complementary role of empathic, individualized communication. The high mean ratings for perceived value and behavioral intention after a single interaction (means of 4.94 and 4.93 of 5, respectively; Section 4.3) are consistent with prior work showing that empathic chatbot responses are rated more highly than terse factual replies (Ayers et al., 2023) and that future-self visualization mitigates present bias and enhances intrinsic motivation (Mönninghoff et al., 2022). Our system operationalizes both: the digital twin renders a concrete, modifiable multi-year trajectory for the child, while the fine-tuned nutrition-guidance LLM communicates it through warm, supportive dialogue grounded in that trajectory. This grounding distinguishes our approach from short, ungrounded chat interventions, which prior evidence cautions do not reliably produce durable behavior change (Entenberg et al., 2023). The thematic analysis of chat content reinforces this interpretation directly: the dominant theme, present in 93% of participants, was a perception that the recommended changes were achievable without excessive burden, and 64% of participants expressed explicit reassurance or reduced anxiety in response to the guidance - evidence that the empathic, individualized framing of the dialogue, and not merely its factual content, shaped how participants received it. Results for the digital-twin self-assessment questionnaire are reported in Section 4.2, and results for the chat questionnaire’s tracking-app and disease-risk-app items are reported in Section 4.3.

An exploratory analysis reported in Section 4.4 found that participants receiving empathic and supportive AI-generated responses reported marginally higher behavioral-intention scores than those receiving more directive responses alone (mean 4.96 vs. 4.79), but this difference was neither statistically significant (*p* = 0.105) nor of meaningful magnitude, corresponding to a negligible effect size (Cliff’s *δ* = 0.09). This result must be read against a pronounced ceiling effect - 114 of 121 participants gave the maximum behavioral-intention rating - which compresses the available variance and limits what any between-group comparison can reveal: a genuine empathy-related effect, if one exists, could be obscured, but the present data equally cannot establish one. The direction of the difference is consistent with prior work suggesting that empathic communication improves user engagement and motivation, yet on this specific point the study is best regarded as hypothesis-generating rather than confirmatory. Future studies with larger cohorts and objective behavioral outcomes should further investigate which specific dialogue characteristics most effectively support behavioral intention and, ultimately, sustained behavior change.

### 5.5 Reframing DOHaD: motivation, not intervention target

A conceptual point warrants emphasis, and the study’s population reinforces it directly. The Developmental Origins of Health and Disease framework motivates attention to the perinatal period, but the epigenetic programming it describes is fixed largely before and around birth and is not the target our intervention seeks to reverse. Because participants in this study are women with a child already under 14 years of age, rather than currently pregnant women, the intervention is inherently postnatal in focus: the locus of intervention is the modifiable postnatal trajectory - the family’s ongoing diet and physical activity - where childhood overweight is known to be reversible when addressed early (Juonala et al., 2011; Bjerregaard et al., 2018). Framing the digital twin’s prediction as a modifiable future rather than a fixed fate is therefore both scientifically accurate and motivationally appropriate, aligning the system’s persuasive logic with what behavior change can actually accomplish.

### 5.6 Limitations

Several limitations should be noted. Foremost among these, the reported accuracy of the nutrition-guidance LLM should be interpreted with particular caution: it rests on a limited evaluation set of only 30 questions and, in the absence of any standardized Japanese benchmark for nutrition counseling, on the judgment of a small three-rater panel rather than an externally validated standard, so the 90% appropriateness figure should be read as promising but preliminary rather than as a definitive estimate of clinical performance. The nutrition-guidance-LLM accuracy evaluation relied on three registered dietitians whose ratings showed substantial-to-almost-perfect agreement (Fleiss’ kappa = 0.86 for the off-the-shelf condition and 0.75 for the SFT condition; Supplementary Table S1), but the panel comprised only three raters and the evaluation set was modest at 30 items; larger, formally quantified multi-rater evaluation - ideally against a Japanese nutrition-specific benchmark of the kind discussed in Section 2.4, which does not yet exist - would further strengthen the accuracy claims. In addition, the 30-item evaluation set was adopted from an existing cardiovascular-nutrition question framework and has not been specifically validated for Japanese child-nutrition counseling, so its coverage of the present use case is indirect. The behavioral outcomes derive from a single-arm, single-session design and from self-report, which captures behavioral intention, acceptability, and perceived change rather than objectively measured, sustained behavior, and intention does not guarantee long-term action. Because the study lacked a control or comparison arm and delivered the digital-twin visualization and the chat consultation consecutively within one session, the relative contributions of the two components cannot be separated, and the observed responses cannot be attributed specifically to the empathic, digital-twin-grounded design as opposed to engagement with any digital tool. The core acceptability and behavioral-intention items exhibited pronounced ceiling effects (most responses at the maximum of the scale), which limited the ability to detect associations between dialogue characteristics and questionnaire outcomes, as seen in the exploratory analysis of Section 4.4. In particular, the protein-related-guidance comparison was severely underpowered because only two participants were classified as not receiving such guidance, and it should not be interpreted substantively. The coding of AI response characteristics in Section 4.4 was likewise not performed in duplicate, and inter-coder reliability was not assessed. Participant background data reported for cohort-level characterization were limited to maternal age and BMI (Table 1); although child sex, birth weight, and related handbook values were entered transiently to generate each digital twin (Section 3.3), they were not retained in structured form, and maternal and child body-composition measures and the extent of missing handbook data were not recorded at all. This constrains characterization of the sample and any subgroup interpretation, and a fuller participant-characteristics table, with these values retained, should accompany a future confirmatory report. The predictive model adopted from the TMM BirThree cohort showed only modest discrimination for adolescence (AUC 0.692–0.720) and has not been externally validated outside that cohort, so trajectory predictions at older ages should be interpreted cautiously; because the model’s inputs were originally collected at 18–23 months of age and were obtained here from participant-reported handbook records without imputation, visualizations for participants with incomplete records are better understood as simulation-based illustrations than as fully individualized predictions. The study was conducted in Gunma Prefecture, Japan, and the sample comprised 121 women who consented to participate in the research, each with a child under 14 years of age; generalizability to other regions, populations, family structures, and healthcare systems remains to be established. Finally, fine-tuning on a focused dataset carries some risk of overfitting and reduced generalization beyond the child-nutrition domain, a possibility not exhaustively tested here.

### 5.7 Future directions

Future work should pursue a randomized, controlled evaluation with an active comparator to isolate the contribution of empathic, digital-twin-grounded dialogue, and should extend the observation window beyond a single interaction to assess durability of behavior change and, ultimately, objective outcomes in the family. Constructing a Japanese, child-nutrition-specific accuracy benchmark - analogous to the Registered Dietitian exam benchmark used in the United States (Azimi et al., 2025) but grounded in the national food composition database and Japanese dietary reference intakes - would allow larger-scale, reproducible certification of clinical reliability beyond the modest three-rater, 30-item evaluation used here. External validation of the predictive model in independent cohorts, and prospective linkage of the digital twin’s predictions to measured childhood growth, would close the loop between prediction and outcome. A larger-sample replication of the exploratory empathic-response analysis reported in Section 4.4, adequately powered to detect an effect of the small magnitude observed here (Cliff’s *δ* = 0.09), would help determine whether the present, non-significant trend reflects a true association or ceiling-effect noise. Finally, combining the complementary strengths of SFT and RAG - internalized domain knowledge for stability, optional retrieval for currency - may offer a hybrid path that preserves self-containment while accommodating guideline updates.

## 6 Conclusion

This study provides preliminary evidence of the operational feasibility and acceptability of a precision-preventive intervention that integrates a cohort-validated digital twin with an empathic, supervised-fine-tuned open-source nutrition-guidance LLM to support behavioral intention toward change in the prevention of childhood obesity, in a sample of 121 women with a child under 14 years of age. Supervised fine-tuning of Llama 3-70B on the national food composition database raised the mean appropriate-response rate from 18/30 (60.0%) to 27/30 (90.0%) as judged by three registered dietitians, reaching the same general order of appropriateness, in an indirect comparison, as that reported for retrieval-augmented approaches, while yielding a more operationally self-contained, locally controllable, and vendor-independent system. The digital-twin visualization and the chat consultation were both rated highly on their core items (means of 4.89–4.94 of 5, standard deviations below 0.4), while interest in a prospective standalone application was more moderate and variable (means of 3.27–4.25, standard deviations of 1.09–1.30). Thematic analysis of chat content showed that 93% of participants perceived the recommended changes as feasible to start gradually. These findings suggest that pairing accurate, individualized prediction with empathic, grounded communication may elicit preliminary motivational responses toward lifestyle change, although the single-arm design cannot isolate this benefit from that of information delivery alone. Framed appropriately - targeting the modifiable postnatal trajectory rather than fixed prenatal programming - such systems offer a scalable, resource-efficient pathway toward the early prevention of childhood obesity and its downstream non-communicable disease burden. Controlled, longitudinal evaluation will be needed to confirm that these early motivational gains translate into durable behavior change and improved long-term outcomes.

## Data Availability

The datasets generated and analyzed during the current study are not publicly available because they contain potentially sensitive participant information and are subject to ethical and institutional restrictions. De-identified data may be made available by the corresponding author upon reasonable request, subject to approval by the relevant ethics committee and applicable institutional regulations.
