# Supplementary material for: Digital twin–supported behavioral intention in mothers of young children to prevent childhood obesity: a large language model–based intervention study
Source: Front Artif Intell. 2026 Aug 6;9:1927993. doi: 10.3389/frai.2026.1927993 (PMC13491048; doi:10.3389/frai.2026.1927993)
Supplement: Supplementary file 1 [file Table_1.DOCX]

**Supplementary Table S1. Per-item appropriateness ratings for the 30-question nutrition evaluation set, by model condition and by each of three registered dietitians (A, B, C).**

| **No.** | **Category** | **Question** | **Off-the-shelf Llama 3-70B** | | | **Llama 3-70B + SFT** | | |
| --- | --- | --- | --- | --- | --- | --- | --- | --- |
|  |  |  | **A** | **B** | **C** | **A** | **B** | **C** |
| 1 | Cooking and food selection | What cooking oils are recommended for a heart healthy diet? | Appropriate | Appropriate | Appropriate | Appropriate | Appropriate | Appropriate |
| 2 | Salt and sodium intake | How can I reduce sodium for a heart healthy diet? | Inappropriate | Inappropriate | Appropriate | Appropriate | Appropriate | Appropriate |
| 3 | Salt and sodium intake | How much sodium should I eat per day on a heart healthy diet? | Inappropriate | Inappropriate | Inappropriate | Appropriate | Appropriate | Appropriate |
| 4 | Popular and specialized diets | Is intermittent fasting a good way to lose weight and healthy for the heart? | Inappropriate | Inappropriate | Inappropriate | Appropriate | Appropriate | Appropriate |
| 5 | Popular and specialized diets | Is the ketogenic diet heart healthy? | Inappropriate | Inappropriate | Inappropriate | Appropriate | Appropriate | Appropriate |
| 6 | Individualization and safety | What should I eat to lower my blood pressure? | Inappropriate | Inappropriate | Inappropriate | Inappropriate | Inappropriate | Inappropriate |
| 7 | The three macronutrients | What kinds of carbohydrates should I be eating as part of a heart healthy diet? | Appropriate | Appropriate | Appropriate | Appropriate | Appropriate | Appropriate |
| 8 | Individual food groups | How many eggs can I eat on a heart healthy diet? | Appropriate | Appropriate | Appropriate | Appropriate | Appropriate | Appropriate |
| 9 | Individual food groups | How much red meat can I eat on a heart healthy diet? | Appropriate | Inappropriate | Inappropriate | Appropriate | Appropriate | Appropriate |
| 10 | The three macronutrients | What are some good snack options that are higher in protein? | Appropriate | Appropriate | Appropriate | Appropriate | Appropriate | Appropriate |
| 11 | The three macronutrients | What are some good plant based protein sources? | Appropriate | Appropriate | Appropriate | Appropriate | Appropriate | Appropriate |
| 12 | Fundamentals of a heart-healthy diet | How many meals a day should I eat for a heart healthy diet? | Appropriate | Appropriate | Appropriate | Appropriate | Appropriate | Appropriate |
| 13 | Individual food groups | What counts as a serving of fruits and vegetables? | Appropriate | Inappropriate | Appropriate | Appropriate | Appropriate | Appropriate |
| 14 | Cooking and food selection | Can I eat pizza on a heart healthy diet? | Appropriate | Appropriate | Appropriate | Appropriate | Appropriate | Appropriate |
| 15 | Individual food groups | How do I incorporate canned and frozen fruits and vegetables into my daily servings? | Appropriate | Appropriate | Appropriate | Appropriate | Appropriate | Appropriate |
| 16 | Fundamentals of a heart-healthy diet | Which foods should I avoid on a heart healthy diet? | Appropriate | Appropriate | Appropriate | Appropriate | Appropriate | Appropriate |
| 17 | Individualization and safety | What diet should I follow to lower cholesterol? | Inappropriate | Inappropriate | Inappropriate | Inappropriate | Inappropriate | Appropriate |
| 18 | Individual food groups | Can I have dairy on a heart healthy diet? | Appropriate | Appropriate | Appropriate | Appropriate | Appropriate | Appropriate |
| 19 | Cooking and food selection | What are some heart healthy breakfast ideas? | Appropriate | Appropriate | Appropriate | Appropriate | Appropriate | Appropriate |
| 20 | The three macronutrients | How much added sugar is okay to consume in a heart healthy diet? | Inappropriate | Inappropriate | Inappropriate | Appropriate | Appropriate | Appropriate |
| 21 | The three macronutrients | What are healthy fats to incorporate in a heart healthy diet? | Appropriate | Appropriate | Appropriate | Appropriate | Appropriate | Appropriate |
| 22 | Individualization and safety | How many calories should I be eating per day to support a heart healthy diet? | Inappropriate | Inappropriate | Inappropriate | Inappropriate | Inappropriate | Inappropriate |
| 23 | Fundamentals of a heart-healthy diet | Which diet is best to prevent heart disease? | Appropriate | Appropriate | Appropriate | Appropriate | Appropriate | Appropriate |
| 24 | Beverages | Can I drink alcohol on a heart healthy diet? | Appropriate | Appropriate | Appropriate | Appropriate | Appropriate | Appropriate |
| 25 | Popular and specialized diets | Should I follow a low carb diet to lower cholesterol? | Inappropriate | Inappropriate | Inappropriate | Appropriate | Appropriate | Appropriate |
| 26 | The three macronutrients | How much protein should I eat per day as a part of a heart healthy diet? | Inappropriate | Inappropriate | Inappropriate | Appropriate | Inappropriate | Appropriate |
| 27 | Beverages | Can I drink caffeine on a heart healthy diet? | Appropriate | Appropriate | Appropriate | Appropriate | Appropriate | Appropriate |
| 28 | Beverages | Can I still drink soda on a heart healthy diet? | Appropriate | Appropriate | Appropriate | Appropriate | Appropriate | Appropriate |
| 29 | Individual food groups | Can I eat nuts on a heart healthy diet? | Appropriate | Appropriate | Appropriate | Appropriate | Appropriate | Appropriate |
| 30 | Individual food groups | How much fish should I be eating on a heart healthy diet? | Inappropriate | Inappropriate | Inappropriate | Appropriate | Appropriate | Appropriate |
|  |  | **Inappropriate responses (n/30)** | 11/30 | 13/30 | 11/30 | 3/30 | 4/30 | 2/30 |

Each model response was independently rated as Appropriate or Inappropriate by three registered dietitians (A, B, C) for two model conditions: the off-the-shelf Llama 3-70B and the supervised fine-tuned (SFT) model. Column totals of inappropriate ratings correspond to the values reported in Table 3 of the main text (off-the-shelf model: 11, 13, and 11 for dietitians A, B, and C; SFT model: 3, 4, and 2, respectively).

The 30-item question set and its eight-category structure were adopted from Parameswaran et al. (2025) [J Med Internet Res 2025;27:e78625] to enable a comparable evaluation; the ratings reported here are those of the present study.
